# Severe Thrombocytosis in a Newborn with Subcutaneous Fat Necrosis and Maternal Chorioamnionitis

**DOI:** 10.1155/2020/5742394

**Published:** 2020-02-21

**Authors:** Mitali Sahni, Pooja Patel, Akila Muthukumar

**Affiliations:** ^1^Pediatrix Medical Group, Sunrise Children's Hospital, University of Nevada, Las Vegas, NV, USA; ^2^Department of Pediatrics, University of Texas Medical Branch, Galveston, TX, USA

## Abstract

**Background:**

Subcutaneous fat necrosis (SFN) is a form of transient panniculitis that presents commonly in infants with a history of perinatal insult, particularly hypothermia. It is characterized by subcutaneous nodules and plaques that appear over bony prominences on cheeks, shoulders, buttock, and thighs. SFN is usually associated with various complications including hypercalcemia, thrombocytopenia, hypertriglyceridemia, and hyperglycemia. *Case Presentation.* We present a unique case of a female infant with a history of maternal chorioamnionitis who presented with SFN at 11 days of life with thrombocytosis. The platelet count decreased during the hospital stay, and thrombocytosis resolved over the course of the next two weeks. She did not have any other hematological or metabolic abnormalities associated with SFN.

**Conclusion:**

Infants with perinatal stress are at increased risk of developing SFN during the first month of life. Infants with a diagnosis of SFN should be monitored closely for various hematological and metabolic abnormalities that can have serious consequences.

## 1. Background

Subcutaneous fat necrosis (SFN) is a form of transient panniculitis that presents in newborns in their first month of life, as multiple erythematous to violaceous, indurated plaques or nodules. It is usually seen over areas of bony prominences and usually resolves with time. It is however associated with various serious metabolic and hematological complications like hypercalcemia, for which the patient should be monitored. The pathogenesis of SFN is not clearly defined in the literature, but it is seen in infants where the blood supply to the fat tissue may be affected due to environmental stress. We present a unique case of SFN who also presented with severe thrombocytosis. SFN has been associated with thrombocytopenia; however, there are no reported cases of SFN with thrombocytosis in the literature.

## 2. Case Presentation

**Figure 1:**
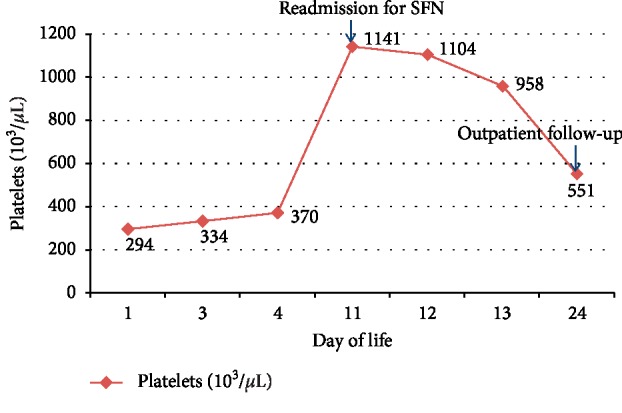
Progression of total platelet count-infant with normal platelet count at birth, thrombocytosis noted on the readmission to hospital for SFN and progressive decrease over the next two weeks.

An eleven-day-old female infant was admitted to the pediatric inpatient floor due to a two-day history of increase in size and number of nodules on the back. She was born at 38 weeks gestational age, to a G1P1 mother via Cesarean section. The perinatal course was complicated by maternal chorioamnionitis; infant underwent a sepsis workup which was negative and received IV antibiotics for 48 hours only. Complete blood count (CBC) done during this period revealed normal CBC indices including a normal total platelet count. She was discharged from the nursery on day of life 5 and was readmitted on day 11 of life for new-onset nodules and increased irritability. On physical examination, there were multiple oval, rubbery, nonmobile, nonfluctuant 1 × 1 cm nodules, palpable at occiput and nape of the neck. Also, a large 4 cm violaceous plaque was found on her back interspersed with multiple, firm, nonmobile nodules. These lesions looked erythematous in some areas and were tender to touch. Initial evaluation revealed an elevated platelet count of 1104 × 10^3^/*μ*L with normal leukocyte count, normal C-reactive protein, and normal blood smear. The platelet count continued to decrease over the course of the hospital stay, and thrombocytosis resolved over the next two weeks.  Figure 1 shows the progression of total platelet count from birth until 4 weeks of age. Our initial differential diagnosis included infections like herpes simplex virus, malignancies like neuroblastoma, infantile myofibromatosis and rhabdomyosarcoma, subcutaneous fat necrosis, and skin necrosis due to protein C/S deficiency.

**Figure 2:**
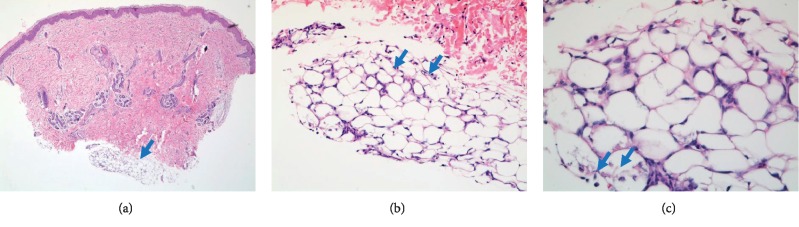
Hematoxylin-eosin stained section of the skin biopsy shows in (a) normal epidermis and dermis with underlying lobular panniculitis (blue arrow) and necrosis of the fat globules in the subcutaneous adipose tissue. (b) The panniculitis (blue arrows) comprised of acute (neutrophils) and chronic (lymphocytes) inflammatory cells and histiocytes. (c) shows many fat cells retained their outline but contain fine, eosinophilic cytoplasmic strands and granules, between which are narrow clefts radiating (blue arrow) from a point near the periphery of the cell.

The infant was started on the IV antibiotics ampicillin and gentamicin along with IV acyclovir that were later discontinued after cultures resulted negative. Retroperitoneal ultrasound done to rule out any abdominal mass was reported to be normal. A punch biopsy of the lesion was obtained, and it showed a focus of inflammatory cells in the fat lobule with focal needle-like spaces, consistent with subcutaneous fat necrosis (Figure 2). The nodules remained stable during the hospital stay, and the infant was discharged on DOL 13. Twelve days after discharge, CBC done at the time of outpatient follow-up indicated resolving thrombocytosis (551 × 10^3^//*μ*L) and stable calcium levels (10.2 mg/dL). Three months after discharge, the large violaceous plaque and skin nodules were mostly resolved.

## 3. Discussion

SFN is a rare form of benign panniculitis seen in the first four weeks of life in term and postterm infants. It is essential to identify it early, as it has been known to cause many complications including hypercalcemia, which can be fatal. The lesions of SFN are characterized by multiple erythematous to violaceous, indurated plaques or nodules most commonly found on the cheeks, buttocks, posterior trunk, and extremities. Of note, they tend to spare the anterior trunk. Many lesions become calcified or fluctuant with liquefied fat. Mild atrophy of the skin may be noticed after resolution [1].

The cause of SFN is not precisely known; however, there are many risk factors associated with it. A systemic review of 16 patients with SFN performed by Mahé et al. identified newborn failure to thrive (12/16), forceps delivery (7/16), maternal high blood pressure (3/10) and/or diabetes (2/10), macrosomia (7/16), exposure to active (4/10) or passive (3/10) smoking during pregnancy, putative or known maternal/paternal or newborn risk factors for thrombosis (5/10), and dyslipidaemia (2/10) as the possible causes [2]. Another case-series review by Burden and Krafchik described birth asphyxia and meconium aspiration as etiological factors [3]. There have been multiple case reports of SFN developing in patients after receiving therapeutic hypothermia [4, 5] or after application of ice bags for management of supraventricular tachycardia [6]. Given its varied associations, the pathogenesis of this disease remains unclear. However, there is a definite association with infants in distress secondary to the maternal and fetal factors noted above.

Diagnosis of SFN is usually made clinically, but sometimes a biopsy can be helpful for confirmation. The cytology can show a spectrum of changes ranging from fat lobules with opaque cytoplasm to necrotic aspirates containing fat cells with needle-shaped crystals, multinucleated giant cells, foamy macrophages, lymphocytes, and neutrophils. We did a skin biopsy, which showed inflammatory cells in the fat lobule with focal needle-like spaces, consistent with the diagnosis of SFN [7].

Most SFN lesions spontaneously resolve in a few months, but many complications may be seen. The most commonly reported complications include thrombocytopenia, hypoglycemia, hypertriglyceridemia, and hypercalcemia (which may sometimes lead to nephrocalcinosis) [1]. In a retrospective study of 30 patients with SFN, authors reported the clinical characteristics and complications in infants with a diagnosis of SFN. The complications seen with SFN in their study cohort included hypercalcemia (19/30), thrombocytopenia (11/30), recurrent disease (4/30), nephrocalcinosis (3/30), hypoglycemia (3/30), calcinosis of the gallbladder (1/30), and hyperlipidemia (1/30) [8]. Hypercalcemia has been attributed to high levels of 1, 25-dihydroxy vitamin D produced by macrophages leading to increased calcium absorption from the gastrointestinal tract and also to calcium release from necrotic adipose tissue [9, 10]. In our case, we did not encounter any of these complications, but we noted a significant thrombocytosis at the time of presentation and which slowly resolved during the hospital stay. To the best of our knowledge, this is the first reported case of SFN where thrombocytosis has been reported. Although thrombocytopenia has been reported in many cases of SFN, the cause of this association is not known. Chen et al. proposed that the thrombocytopenia was due to the local sequestration of platelets in the subcutaneous tissue [11]. We propose that the thrombocytosis can be attributed to the reactive inflammatory response of SFN. However, the increased number of platelets could have led to increased sequestration in the subcutaneous tissue, thereby worsening the adipose tissue necrosis.

Although SFN is a relatively benign condition, it has been associated with many hematological and metabolic abnormalities that require follow-up and at times interventions. Since we do not know the pathogenesis behind this phenomenon, any new association noted can be helpful to understand this disease better. Infants diagnosed with SFN should be monitored for hematological and electrolyte abnormalities periodically. Most lesions resolve spontaneously over time, but monitoring for complications is suggested to avoid adverse outcomes.

## Abbreviations

SFN: Subcutaneous fat necrosis
CBC: Complete blood count
DOL: Day of life.
